# A Mouse Mutation That Dysregulates Neighboring *Galnt17* and *Auts2* Genes Is Associated with Phenotypes Related to the Human AUTS2 Syndrome

**DOI:** 10.1534/g3.119.400723

**Published:** 2019-09-25

**Authors:** P. Anne Weisner, Chih-Ying Chen, Younguk Sun, Jennifer Yoo, Wei-Chun Kao, Huimin Zhang, Emily T. Baltz, Joseph M. Troy, Lisa Stubbs

**Affiliations:** * Carl R. Woese Institute for Genomic Biology,; † Neuroscience Program,; ‡ Department of Cell and Developmental Biology, and; § Illinois Informatics Institute, University of Illinois at Urbana-Champaign, Urbana IL 61802

**Keywords:** Neurological development, Autism, Translocation, Gene regulation, Syndromic phenotypes

## Abstract

*AUTS2* was originally discovered as the gene disrupted by a translocation in human twins with Autism spectrum disorder, intellectual disability, and epilepsy. Since that initial finding, *AUTS2*-linked mutations and variants have been associated with a very broad array of neuropsychiatric disorders, sugg esting that *AUTS2* is required for fundamental steps of neurodevelopment. However, genotype-phenotype correlations in this region are complicated, because most mutations could also involve neighboring genes. Of particular interest is the nearest downstream neighbor of *AUTS2*, *GALNT17*, which encodes a brain-expressed N-acetylgalactosaminyltransferase of unknown brain function. Here we describe a mouse (*Mus musculus*) mutation, T(5G2;8A1)GSO (abbreviated 16Gso), a reciprocal translocation that breaks between *Auts2* and *Galnt17* and dysregulates both genes. Despite this complex regulatory effect, 16Gso homozygotes model certain human *AUTS2*-linked phenotypes very well. In addition to abnormalities in growth, craniofacial structure, learning and memory, and behavior, 16Gso homozygotes display distinct pathologies of the cerebellum and hippocampus that are similar to those associated with autism and other types of *AUTS2*-linked neurological disease. Analyzing mutant cerebellar and hippocampal transcriptomes to explain this pathology, we identified disturbances in pathways related to neuron and synapse maturation, neurotransmitter signaling, and cellular stress, suggesting possible cellular mechanisms. These pathways, coupled with the translocation’s selective effects on *Auts2* isoforms and coordinated dysregulation of *Galnt17*, suggest novel hypotheses regarding the etiology of the human “AUTS2 syndrome” and the wide array of neurodevelopmental disorders linked to variance in this genomic region.

Autism Susceptibility Candidate 2 (*AUTS2*) was originally identified as the gene disrupted by reciprocal translocation in human twins with Autism spectrum disorder (ASD), intellectual disability (ID), and epilepsy (Sultana *et al.* 2002). Since that first report, hundreds of mutations or variants in the *AUTS2* genomic region have been identified, most of which correspond to translocations, duplications, deletions or other types of genomic rearrangements. Detailed analyses of genotype-phenotype associations have revealed ID and developmental delay as core features of the “AUTS2 syndrome”. However, other phenotypes, including the stereotypic behaviors and abnormal social development characteristic of ASD, feeding difficulties, reduced postnatal growth, epilepsy, microcephaly and characteristic craniofacial abnormalities are also observed in substantial subsets of patients (Beunders *et al.* 2013; Beunders *et al.* 2016). In addition to the syndromic phenotypes associated with genome rearrangements, *AUTS2*-linked single nucleotide polymorphisms (SNPs) have been associated with Attention Deficit Hyperactive Disorder (Elia *et al.* 2010), dyslexia (Girirajan *et al.* 2011), schizophrenia (Zhang *et al.* 2014a), bipolar disorder (Hamshere *et al.* 2009), depression (Myung *et al.* 2015), language disorders (Chen *et al.* 2017; Amarillo *et al.* 2014) and addiction (Kapoor *et al.* 2013; Chen *et al.* 2016; Chen *et al.* 2013). Association with this very wide range of psychiatric disorders suggests that mutations in this region disrupt the functions of genes with central roles in neurodevelopment.

Most attention has focused on the *AUTS2* gene itself, a complex locus spanning more than 1.5 million base pairs (Mbp) that generates distinct protein isoforms from several alternative promoters. Patients carrying two rare exonic deletions in the longest *AUTS2* isoform have been studied in depth, and display central AUTS2 syndrome features including ID and developmental delay as well as ASD, feeding difficulties, growth deficits, microcephaly, and craniofacial abnormalities, supporting the importance of *AUTS2* in these phenotypes (Beunders *et al.* 2015). Studies in *Drosophila* (Schumann *et al.* 2011), zebrafish (Oksenberg *et al.* 2013) and mice (Hori *et al.* 2015; Hori *et al.* 2014; Gao *et al.* 2014) have further demonstrated important roles for *Auts2* in neurogenesis and neuron differentiation. In particular, the mouse studies have revealed that cytoplasmic AUTS2 protein isoforms control the formation of lamellipodia and filopodia, thus impacting neuronal migration and neurite formation (Hori *et al.* 2014), whereas nuclear AUTS2 serves as a co-factor in a novel PRC1gene regulatory complex (Gao *et al.* 2014). Global *Auts2* loss-of-function (LOF) is lethal in homozygous form, associated with grossly disrupted cortical layering and neurite extension deficits (Hori *et al.* 2014). On the other hand, heterozygotes are viable with decreased exploratory activity, reduced anxiety-related behaviors, developmental delay and memory impairments (Hori *et al.* 2015). Furthermore, mice homozygous for a conditional knockout affecting only the longest *Auts2* isoform are viable but small, with abnormal vocalization and delayed development of righting reflexes (Gao *et al.* 2014).

Despite these important clues and an intensive focus on human patients over the past several years, many questions regarding genotype-phenotype relationships in this genomic region remain unanswered. The complexity added by the existence of multiple *AUTS2* isoforms is clearly a significant part of this puzzle. Additionally, most mutations correspond to genomic rearrangements that could possibly affect the expression of neighboring genes. In this regard, the immediate downstream neighbor of *AUTS2*, *GALNT17* (previously named *WBSCR17*), is of particular interest. *GALNT17* encodes a brain-expressed N-acetylgalactosaminyl transferase (GalNAcT) that, like *AUTS2*, regulates the formation of lamellipodia, with impact on cell adhesion and motility (Nakayama *et al.* 2012). However, *Galnt17* mutants have not been examined and virtually nothing is known about the expression or functions of this GalNacT protein in neurons or the brain.

Here we present the characterization of a novel laboratory mouse mutation identified in a genetic screen, T(5G2;8A1)GSO (or 16Gso), a reciprocal translocation breaking between *Auts2* and *Galnt17* that, presumably through the disruption of regulatory interactions, leads to the mis-expression of both genes. We investigated the behavioral and neuropathological phenotypes of mutant animals and integrated these data with gene expression analysis to develop new hypotheses regarding the functions of both genes, and new clues to the neurodevelopmental correlates of AUTS2 syndrome phenotypes.

## Material and Methods

### Animals and Tissue Collection

The generation of 16Gso mice has been described previously (Elso *et al.* 2008; Weisner 2015). The genotypes studied here were in mice maintained on a C57BL/6 X C3H/He F1(B6C3) genetic background. In order to efficiently collect animals of each genotype for testing, 16Gso homozygotes (simplified as 16GsoT/+) were generated by breeding homozygous parents; heterozygotes were collected separately in crosses involving 16Gso homozygotes (abbreviated as 16GsoT/T) and wild type (WT) mice. Animals were housed under standard conditions (12h light/dark cycle, group-housed). This study was carried out in strict accordance with the recommendations in the Guide for the Care and Use of Laboratory Animals of the National Institutes of Health. The protocol was approved by the Institutional Animal Care and Use Committee of the University of Illinois (Animal Assurance Number: A3118-01; approved IACUC protocol number 18240).

### Mapping and sequencing the 16Gso translocation breakpoint

Metaphase chromosome FISH was performed as previously described (Elso *et al.* 2004). BACs from the RP23 and RP24 BAC libraries were used as template for FISH probes. The breakpoint region was narrowed by determining BACs that were above, below or split by the breakpoint region by co-hybridization with a control BAC used to mark proximal regions of chr5 and chr8. The analysis identified two overlapping chr5 BAC clones (RP23-7a8 and RP23-283H6) and two chr8 clones (RP24-234k3 and RP24-234a5) that spanned breakpoint sites. We chose ∼500 kb intervals surrounding the regions of BAC overlap for hybrid selection and deep sequencing. Both hybrid selection and sequencing were performed by Otogenetics Inc. (Atlanta), and approximately 30X depth of paired-end Illumina sequence reads from two 16GsoT/T and two WT animals for each region was returned for our analysis. We mapped reads to the mm9 genome build using Bowtie software (Langmead *et al.* 2009), and identified sets of paired ends that mapped to different chromosomes. Of those reads, we found clusters of end-pairs with one end mapping to a region of chr5 and the other end mapping to chr8; these were the only endpairs to map repeatedly to any genomic region. Finally, we developed primers from regions of chr5 and 8 covered by the clustered clones, and used PCR to generate and sequence chr5;8 and chr8;5 breakpoint spanning regions. New primer sets were designed around the breakpoint site for reliable PCR genotyping.

### Mouse Phenotypic tests

WT, 16GsoT/+ and 16GsoT/T pups were tested for developmental delay at postnatal day 5 (P5), P6, P7, P8 and P9, in the form of the righting reflex; the same animals were tested for freezing, repetitive behavior and novel object recognition tasks at 6-8 weeks old. The weight for each animal was also measured at P5, P7, P9 and P35.

#### Righting reflex:

righting reflex test was modified from a previous study (Castelhano-Carlos *et al.* 2010). The pups were flipped and the time for them to turn back to their original position was recorded. Different degrees of performance on turning back were assigned to specific score for each day of the testing, score 0 = failed to turn back within 15 sec; score 1 = pups turned back between 10 to 15 sec; score 2 = pups successfully made right turn within 10 sec for the first time; score 3 = pups successfully made right turn within 10 sec for the second time.

#### Freezing behavior:

the freezing time was measured when the mouse was placed in a novel environment, a 35 cm × 30 cm paper box, for 10 min.

#### Repetitive behavior (grooming):

The average grooming time per episode in 10 min interval was measured, when mice were placed in an empty box as used in freezing tests.

#### Novel object recognition (NOR):

NOR tasks were followed as previously described (Botton *et al.* 2010). Tasks included three sessions: habituation, training and retention. In the habituation session, the mouse was placed in an empty 35 cm × 30 cm paper box for 10 min, and returned to its home cage. The training session was held 24 hr after the habituation session. In the training session, two novel objects (Legos were used in this experiment) were placed in the box, and the exploratory behavior was monitored for 10 min. In the retention session, which was carried out 24 hr after the training session, one of the familiar Legos from training session was exchanged for a new Lego, and the mouse was allowed to explore both objects for 5 min. Exploratory behavior was defined as sniffing or touching the objects. The exploratory index was calculated as exploration time on novel object to total exploration time, as a percentage.

#### Home cage activity:

Home cage activity was also monitored for adult animals between three and seven months of age. Activity was monitored continually for 7 days, during which animals were singly housed, using overhead cameras under white or red light and Topscan software to track animal movement.

#### Skull Measurements:

mouse skulls were cleaned of skin and viscera, and the snout was measured from the distal tip to the anterior notch of the frontal process, lateral to the infraorbital fissure. Measurements were also taken from the distal tip of the snout to the bregma, the midline intersection of the frontal and parietal bones, and to the intersection of the interparietal bones and occipital bones at the base of the skull.

### Immunohistochemistry

Isolated tissues were fixed in fresh 4% paraformaldehyde, embedded in paraffin, and cut into 5-6 µm sections using a Leica RM2155 microtome and Super Plus charged slides. Cerebellum (CB) slices were sectioned into sagittal sections, and hippocampus (HC) sections were in coronal orientation. For IHC, the primary antibody was used at optimal ratio in Antibody Diluent Reagent Solution (Life Technologies) and incubated overnight at 4°. Slides were then washed and incubated with secondary antibody (1:200) in antibody diluent for 1 hr at room temperature. Slides were further washed and stained with Hoecsht 3342 and scanned under Zeiss LSM 710 Confocal Microscope for further analysis. Primary antibodies: MAP2 (Abcam ab5392, 1:300), AUTS2 (Sigma HPA000390, 1:200), WBSCR17 (LSBio LS-B14501, 1:250), Calbindin D-28K (Sigma C9848, 1:300), Calretinin (Santa Cruz sc-365989, 1:300), CNPase (Millipore MAB326, 1:250), DRD2 (Millipore AB5084P, 1:200), GFAP (Invitrogen 180063, 1:300), GAD 65/67 (Santa Cruz sc-365180, 1:200), Tbr2 (Abcam ab23345, 1:100), cFos (Abcam ab190298, 1:200); Secondary antibodies (Thermo-Fisher Scientific): Goat anti-Mouse IgG (Alexa Fluor 488, A11001), Goat anti-Rabbit IgG (Alexa Fluor 594, A11012), Donkey anti-Rat IgG (Alexa Fluor 594, A21209).

### Histopathology

Paraffin-embedded slides were stained with hematoxylin and eosin (H&E). For CB, sections were taken from the midline, spaced at 600 micrometer intervals in adults, 300 micrometer intervals in P14 animals, and 200 micrometer intervals in P7 animals. Purkinje cells (PCs) were counted by two independent researchers who were blinded to the genotypes of the animals (n = 3 of each genotype and age), and who were trained to identify cells based on size and morphology. For the hippocampal cell density experiment, the cells were counted in randomly assigned 90 µm × 90 µm squares in dentate gyrus, CA3, and CA1 on 20X magnification, from slides taken from dorsal regions of the HC (n = 3 mice of each genotype and age). The thickness of the cell layer was measured through NanoZoomer Digital Pathology software.

### RNA-seq analysis

CB and HC tissues were collected from P14 and P35 WT and 16GsoT/T, and RNA was purified using Trizol followed by 45 min of RNase-free DNase I treatment (NEB) and RNA Clean & Concentrator Kit (Zymo Research). The quality of RNA was tested by Agilent BioAnalyzer, and Illumina libraries were generated using the KAPA Stranded mRNA-Seq kit with MRNA Capture Beads (Kapa Biosystems). Sequencing was performed on Illumina Hi-Seq 2000 sequencers. Reads were mapped using Tophat2 software (Kim *et al.* 2013), transcripts quantified using HT-seq count (Anders *et al.* 2015), and differential expression determined using the edgeR package (Robinson *et al.* 2010) as we have previously described (Saul *et al.* 2017). Differentially expressed genes determined with fdr ≤ 0.05 were analyzed for functional enrichment using DAVID (Huang da *et al.* 2009).

### Quantitative RT-PCR (qRT-PCR)

RNA was prepared from snap frozen tissues and purified using Trizol (Invitrogen) extraction followed by 45 min of RNase-free DNase I treatment (NEB) and RNA Clean & Concentrator Kit (Zymo Research). cDNA was then prepared using a total of 2 ug RNA in a 20 µl reaction with Random Primer Mix (NEB) and M-MuLV Reverse Transcriptase (NEB). qRT-PCR was then carried out using custom-designed primers sets specific for *Auts2* isoform transcripts (Table S2) with Power SYBR Green PCR master mix (Applied Biosystems) in a final volume of 10 μl. Reactions were run on an ABI 7900 thermocycler for 40 cycles with an annealing temperature of 60°. Expression values were normalized relative to the Pgk1 control in each sample and compared using standard methods (Livak and Schmittgen 2001).

### Data availability

All data and resources reported in this manuscript are publicly available. Sequencing data reported in this paper has been deposited into the GEO database under accession number GSE132684. The results of sequence analysis are presented in Table S2 and full listing of enriched functional categories from DAVID analysis in Table S3. Oligonucleotide primers used for qRT-PCR are list in Table S4. Data from behavioral tests and represented as graphs in this manuscript are presented in Table S1, and H&E stains of 16Gso T/T and WT cortex are presented in Fig. S1. Sequence confirming the expression of novel *Auts2* isoform T6, which is identical to that in an ENSEMBL model and mouse ESTs as reported, is also provided in Supplemental Text. Supplemental material available at FigShare: https://doi.org/10.25387/g3.9887540.

## Results

### Genetic and molecular analysis of the 16Gso mutation

The 16Gso mutation arose in a genetic screen of the offspring of males exposed to *bis*-acrylamide, a treatment that efficiently generates reciprocal translocations in late stage spermatocytes (Stubbs *et al.* 1997). Karyotype analysis confirmed that the animals carried a reciprocal translocation affecting chromosome 5 (chr5) band G2 and chromosome 8 (chr8) band A1 (Stubbs *et al.* 1997). Heterozygotes for the 16Gso translocation (hereafter abbreviated as 16GsoT/+ for simplicity) were overtly normal, and although most 16Gso homozygotes (16GsoT/T) survived to adulthood and were fertile, they were generally smaller than wild type (WT) littermates, and were otherwise frankly abnormal in appearance and behavior.

To map the 16Gso breakpoint more precisely, we used methods described previously (Elso *et al.* 2004; Robinson *et al.* 2005; Elso *et al.* 2013; Bolt *et al.* 2014), selecting bacterial artificial chromosome (BAC) clones from chr5 and chr8 to use as probes in fluorescent *in situ* hybridization (FISH). This narrowed the breakpoint to BAC overlap regions of approximately 130 kb on chr5 (chr5:131,812,581-131,945,233 in NCBI build 37) and ∼193kb on chr8 (chr8:12,244,884-12,437,596). Next, we enriched DNA spanning approximately 500 kb around both breakpoint regions with hybrid-selection based-methods and focused on paired ends mapping to different chromosomes - with one end on chr5 and the other on chr8 – to pinpoint the location of the breakpoint. Finally, we used PCR to confirm, isolate and sequence *trans*-breakpoint DNA fragments to reveal the structure of the translocation breakpoints.

**Figure 1:**
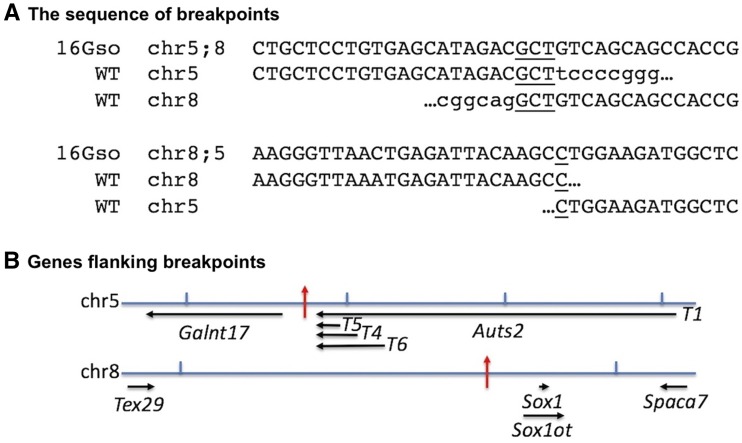
Sequence and genomic map location of the 16Gso translocation breakpoints. (A) Sequences obtained by PCR across the 16Gso breakpoint aligned with the WT sequence at the same genomic sites. Sequences that align between WT chr5 and chr8 and the mutant DNA sequences are shown in caps, while lower case letters represent WT sequences beyond the breakpoint site. The alignment revealed the deletions on both translocated chromosomes (underlined), involving one copy of a homologous triplet (GCT) shared by the two WT chromosomes in the translocated chr5;8, and deletion of a single C at the chr8;5 breakpoint site. (B) The breakpoint-flanking sequences mapped between *Galnt17* and *Auts2* genes on chr5, and upstream of *Sox1* on chr8 (red arrows on each chromosome map). Genes are drawn to scale within each map; arrows indicate direction of transcription for each gene. For *Auts2*, transcripts T1, T4, T5, and T6 are labeled at the locations of their promoters. Above both maps, vertical lines demark a genomic distance of 500 kb.

The sequence of these breakpoint junction fragments confirmed the fusion of chr5 and chr8 sequences in 16Gso DNA (Figure 1A). The 16Gso breakpoint is located about 70kb upstream of *Sox1* on chr8 (mm9 coordinates chr8:12325249); on chr5 it is situated between *Galnt17* and *Auts2*, ∼72 kb upstream and 60 kb downstream of those genes, respectively (chr5:131854998) (Figure 1B, red arrows). Like other germline translocations we have sequenced (Elso *et al.* 2008; Elso *et al.* 2013; Bolt *et al.* 2014; Elso *et al.* 2004; Robinson *et al.* 2005), the T(8;5) and T(5;8) 16Gso breakpoints are simple in structure, with small deletions (6 and 8 bp on chr 5 and chr8, respectively) and short (1-3 nucleotide) duplications at the breakpoint fusion sites, the classic signature of non-homologous end-joining repair. Aside from these short deletions and duplications at the breakpoint site, we saw no evidence of further rearrangement in the DNA sequence or in extensive FISH and genomic PCR across both breakpoint regions.

### Initial phenotypic characterization of 16Gso mutants

**Figure 2:**
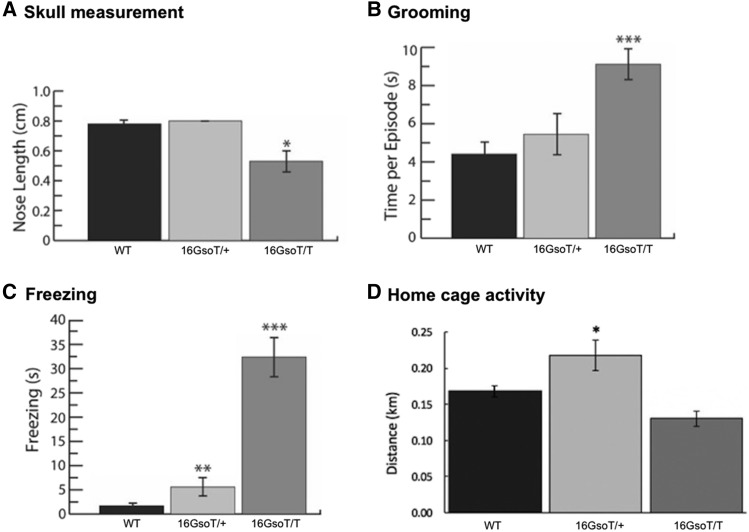
Phenotypic features in 16GsoT/T mutants. (A) Skull measurement: the length from base to tip of the nose was significantly decreased in 16GsoT/T (n = 3) compared to WT (n = 4) and 16GsoT/+ mice (n = 3). (B) Repetitive grooming behavior: the average grooming time for each episode within a 10 min experimental period was significantly increased in 16GsoT/T (n = 9) compared to WT (n = 11) and 16GsoT/+ (n = 12). (C) Freezing behavior: 16GsoT/T (n = 14) froze more in the novel environment compared to WT (n = 10) or 16GsoT/+ (n = 14) in 10 min experimental period. (D) Home cage activity: the average distance traveled (quantified through Topscan software) was increased in 16GsoT/+ (n = 10), but there was no significant difference between WT (n = 9) and 16GsoT/T (n = 5) animals. Skull measurement: ANOVA, *P < 0.05. Grooming and freezing: t- Test, **P < 0.01; ***P < 0.001. Home cage activity: Tukey’s test, *P < 0.05.

16GsoT/T mice were selected for study because they displayed several obvious physical and behavioral phenotypes, including a relatively shortened skull due specifically to reduced length of the snout (Figure 2A; Table 1; Table S1). In addition, 16GsoT/T adults displayed stereotypic behaviors, such as repetitive self-grooming (Figure 2B). Mature 16GsoT/T animals froze in place for extended periods in response to even minor stressors such as handling or after placement in novel environments, suggesting increased levels of anxiety (Figure 2C). Furthermore, some older 16GsoT/T adults (>6 months old) displayed obvious seizures under stressful (*e.g.*, testing) conditions. In contrast, younger 16GsoT/T animals never displayed this seizure phenotype, and none of the many 16GsoT/+ mice we have observed have exhibited seizures at any age.

**Table 1:** Phenotypes detected in 16GsoT/T mice compared with those identified in published KO mutants, AUTS2 syndrome, and ASD human patients

Phenotype	16Gso T/T	*Sox1 −/−**^a^*^,^*^b^*	Auts2−/−*^c^*	Auts2-/+*^d^*	Auts2-T1−/−*^e^*	Related phenotypes in *AUTS2* or ASD patients*^f^*
Small size, low postnatal growth	+	—	nr	+	+	Low birth weight, short stature
Delayed righting	+	nr	.	+	nr	Developmental delay
Learning and memory deficits	+	—	.	+	nr	Intellectual disability
Cortical layering defects	—	—	+	*-*	nr	Intellectual disability
Abnormal skull/nose length	+	—	nr	nr	nr	Dysmorphic features
Freezing behavior, anxiety	+	+	.	nr	nr	Adverse response to novelty, anxiety
Reduced exploratory activity	+	nr	.	+	nr	Intellectual disability
Abnormal nesting	+	nr	.	nr	nr	Abnormal social behavior
Repetitive grooming behaviors	+	—	.	nr	nr	Stereotypic behavior, obsessive compulsive behavior
Seizures	+	+	.	nr	nr	Epilepsy*^f^*^,^*^g^*
Abnormal Purkinje Cell differentiation	+	—	.	nr	nr	ASD*^h^*
Abnormal Purkinje Cell dendrites	+	—	.	nr	nr	ASD*^h^*
Purkinje Cell loss	+	—	.	nr	nr	ASD*^h^*
Abnormal numbers and packing of hippocampal neurons	+	—	.	nr	nr	ASD*^h^*

+ in each column denotes that the phenotype has been observed; - denotes that the phenotype was investigated but not detected; nr = not reported; . = not applicable (animals die around the time of birth).

Data taken from ^*a*^Malas *et al.*, 2003

b Sottile *et al.*, 2006

c Hori *et al.*, 2014

d Hori *et al.*, 2016

e Gao *et al.*, 2014

f Summarized from (Beunders *et al.* 2013) unless otherwise noted

g (Sultana *et al.* 2002)

h (Amaral *et al.* 2008).

In contrast to these abnormal behaviors under stress, 16GsoT/T mice displayed mostly normal behaviors when left undisturbed in their home cage, with no differences from WT animals in overall levels of activity throughout the day (Figure 2D). In their home cages, the mutants can be observed grooming cagemates and sleeping in pairs in a manner not significantly different than WT mice. For the above-mentioned phenotypes and others described below, we tested males and females separately and noted some quantitative and age-dependent differences, but males and females followed very similar trends. The single exception was repetitive self-grooming, which male but not female 16GsoT/T animals displayed (Table S1).

16GsoT/+ mice were normal for most of the 16GsoT/T phenotypes we documented, supporting a primarily recessive inheritance. However, the fact that 16GsoT/+ male (but not female) animals display a slight increase in freezing behavior (Figure 2C) and home cage activity (Figure 2D**)** indicated the possibility of subtle semi-dominant effects.

### Phenotypic comparisons to mutations in specific breakpoint-spanning genes

We next sought to test 16Gso mutants for phenotypes already associated with the breakpoint-flanking genes. *Galnt17* mutants have not been characterized to date in any species, and phenotypes related to *Galnt17* mutations could not be considered here. However, knockout (KO) mutations of both *Sox1* and *Auts2* have been reported. *Sox1* KO homozygotes display seizures beginning at late juvenile stages (age 4-6 weeks), and show evidence of freezing as well as other anxiety-related behaviors – all suggestive of phenotypes already noted for 16GsoT/T mice (Table 1). However, the behavior of *Sox1* KO animals is otherwise not different from wild type animals (Malas *et al.* 2003).

**Figure 3:**
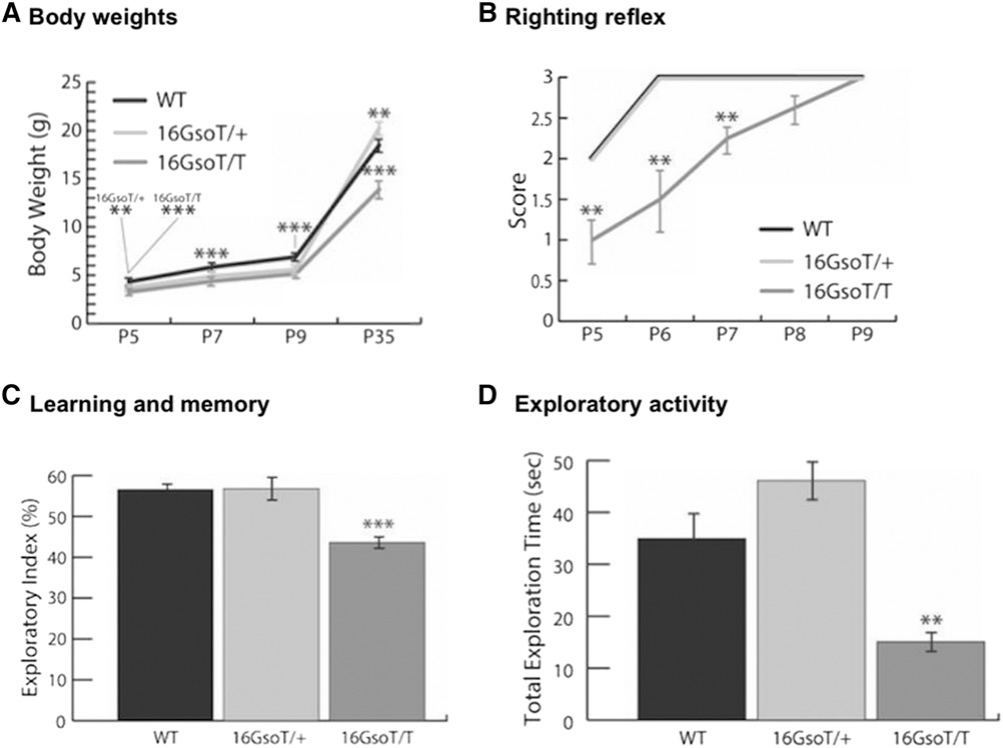
16GsoT/T mutants show lighter weight, developmental delay, impaired learning and memory, and reduced exploratory activity. In each case, 16GsoT/T and 16GsoT/+ were compared to WT age-matched controls. (A) Body weight was measured at P5, P7, P9 and P35 for each genotype (WT n = 9-17; 16GsoT/T n = 9-19; 16GsoT/+ n = 11-21 depending on age). Both 16GsoT/+ and 16GsoT/T weighed significantly less than WT at P5, P7, and P9. At P35, 16GsoT/T was lighter than WT, but 16GsoT/+ animals weighed slightly more than WT. (B) Developmental delay: The 16GsoT/T mice (n = 14) showed delayed development of the righting reflex, while there was no difference between WT (n = 14) and 16GsoT/+ (n = 14) males. (C) Novel object recognition test revealed that 16GsoT/T males (n = 8) showed impaired learning and memory, while WT (n = 8) and 16Gs T/+ animals (n = 9) have normal learning and memory. (D) Exploratory activity was determined in the training session of the NOR test, and showed significantly lower levels of object exploration by 16GsoT/T mice. The number of animals tested in this experiment are identical to that for (C). P-values determined with the t-Test, **P < 0.01; ***P < 0.001.

Additionally, *Auts2* KO mice – including one affecting all transcript isoforms (Hori *et al.* 2014) and another specifically targeting the longest *Auts2* isoform (referred to here as T1, Figure 1; (Gao *et al.* 2014)) have been characterized in some detail. These KO animals display growth retardation, delayed righting reflex, and learning deficits, all of which are relevant to human AUTS2 syndrome phenotypes (Table 1). To quantitate these phenotypes in 16Gso mutants, we completed three additional tests: (1) body weight and length as a function of age, (2) developmental timing of the righting reflex, and (3) novel object recognition (NOR) test, which utilizes the natural exploratory behavior of mice to quantify learning and object recognition. The results confirmed that 16GsoT/T animals were significantly smaller than WT controls from juvenile stages through adulthood as measured by both weight (Figure 3A) and overall body length (Table S1). Furthermore, we found a significant delay in development of the righting reflex in 16GsoT/T pups (Figure 3B). In the NOR test, we found that 16GsoT/T mice showed significant deficits in learning and memory (Figure 3C**)**, and lower levels of object exploration during the training phase compared to WT controls (Figure 3D). Except in the case of body weight at juvenile stages, 16GsoT/+ animals were not significantly different than WT confirming predominantly recessive inheritance (Figure 3A-D**).** Thus, 16GsoT/T mice are similar to *Auts2* KO heterozygotes in displaying small body size, delayed righting, lower levels of exploratory activity, and deficits in the NOR test, suggesting a possible role for *Auts2* loss-of-function in these phenotypes.

### Normal cortical layering, but subtle hippocampal abnormalities and clear cerebellar pathology in 16Gso adults

To ask whether these 16GsoT/T behavioral phenotypes might be associated with obvious brain pathologies, we examined hematoxylin and eosin (H&E)-stained sectioned brains of adult 16GsoT/T and age-matched WT animals. We found marked differences in the structure of hippocampus (HC) and the cerebellum (CB) of the 16GsoT/T mice, two brain regions closely tied to ASD, ID, and neuropsychiatric pathology. In contrast to *Auts2* KO animals (Hori *et al.* 2014), we observed no obvious pathologies in layering or structure of the cortex (Fig. S1), and no obvious malformations elsewhere in mutant brains. Details of the cerebellar and hippocampal pathologies are detailed further as follows.

#### Abnormal development of cerebellar Purkinje cells and Bergmann Glia, and age-dependent Purkinje cell loss:

**Figure 4:**
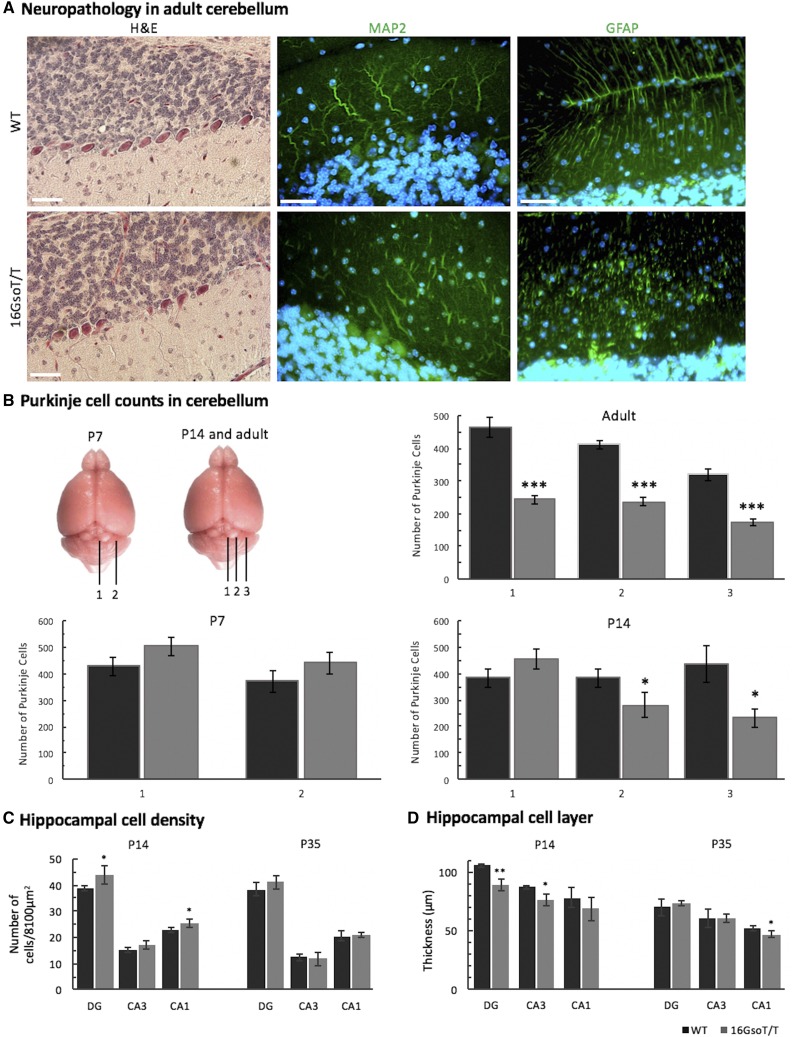
Cerebellar and hippocampal pathology in 16GsoT/T mutants. (A) H&E staining showed that PCs are fewer in number and irregularly spaced in adult 16GsoT/T CB as compared to WT; MAP2 staining reveals that the 16GsoT/T PCs have sparse, irregularly branching dendritic trees, and GFAP staining highlights structural abnormalities of the Bergmann glia. (B) The number of PCs was quantified at different positions throughout the CB at different stages of development (P7, P14 and adult) in WT (black) and 16GsoT/T mutants (gray). Position 1 is at the midline, 1 and 2 each are 200 microns apart at P7, and 1, 2 and 3 each are 300 microns apart at P14, and 600 microns apart in adult. (C) Cell density in DG, CA3 and CA1 was quantified in WT (black) and 16GsoT/T (gray) at P14 and P35. 16GsoT/T has significantly increased cell number in DG and CA1 at P14. (D) Thickness of cell layer was measured in DG, CA3 and CA1 in WT (black) and 16GsoT/T (gray) at P14 and P35. n, WT = 16GsoT/T = 3 males at P7 and P14; WT = 16GsoT/T = 4-5 males in adult. ANOVA, *P < 0.05; **P < 0.01; ***P < 0.001. Scale bar = 50 μm.

First and most obvious, as revealed by H&E-stained sections, mature 16GsoT/T adults had fewer cerebellar Purkinje cells (PCs), ranging from 52–58% of the number found in WT mice; the cells were also irregularly spaced throughout the PC layer (Figure 4A, B). The adjacent granule cell layer was identical in thickness and cell morphology to WT animals (data not shown). To determine the developmental time course of this cerebellar pathology, we then examined H&E stained sections at postnatal days 7 (P7), 14 (P14) and adult. We saw no difference in PC numbers or structure at P7. However, small but significant reductions in PC number were observed in lateral sections of the 16GsoT/T CB at P14, and became severe in adult (Figure 4B).

Immunohistochemistry (IHC) with the MAP2 antibody showed PCs in WT adults to be aligned neatly along the interface between granule cell and molecular layers, extending elaborate dendritic trees with thick primary branches. In contrast, 16GsoT/T PCs were not aligned, and lacked the thick primary branches, with more disorganized dendritic trees (Figure 4A). In contrast to WT animals, PCs in 16GsoT/T mice showed fragmented, disrupted MAP2 staining, indicating abnormal dendritic morphology. Additionally, the GFAP antibody showed radial glial processes of Bergmann glia, which form a scaffold for PC dendrites, also appeared abnormal in 16GsoT/T CB, lacking the organized, linear processes seen in WT sections (Figure 4A). This pathology was first observed at P14 (not shown), but also documented at later stages **(**Figure 4A**).** No pathology was documented in 16GsoT/+ mice at any developmental stage (not shown).

Together these data indicated that in 16GsoT/T mice, PCs are generated in normal numbers and migrate to their final location, but do not develop normally and are lost as the animals age. This pathology is coupled to abnormalities of the Bergmann glia, the development of which is intimately linked to that of PCs (Chrobak and Soltys 2017; Yamada and Watanabe 2002).

As mentioned, other than microcephaly, brain pathology has not been reported for human AUTS2 patients. However, PC loss is the most common brain pathology observed in postmortem analysis of ASD patients (Amaral *et al.* 2008; Pierce and Courchesne 2001; Varghese *et al.* 2017); PC loss is also associated with behavioral rigidity (Dickson *et al.* 2010) and repetitive behaviors in mice (Martin *et al.* 2010). Therefore, the 16GsoT/T PC phenotype is highly relevant to the clinical manifestations of ASD. However, this pathology has not been reported to be associated with any of the breakpoint-linked genes. Specifically, CB has been examined and is normal in *Sox1* KO homozygotes (Sottile *et al.* 2006) and cerebellar abnormalities have not been reported for *Auts2* KO animals of either type (Table 1). Focused studies with these animals or with *Galnt17* mutants could potentially resolve this discrepancy.

#### Abnormal cell numbers and densities in the hippocampal dentate gyrus and Ammon’s horn:

In the P14 16GsoT/T HC, we identified a second set of cellular pathologies with excellent potential to explain certain 16GsoT/T behavioral traits. These included abnormalities in cell layer size, cell numbers, and neuron cell density across the dentate gyrus (DG) and Ammon’s Horn region cornu ammonis 1 and 3 (CA1, CA3). Each region displayed distinct structural phenotypes and different trajectories over the postnatal time course (Figure 4C, D**)**.

Specifically, in P14 16GsoT/T mutants, DG granule cells (GCs) and CA1 pyramidal cells (PyCs) were unusually densely packed; in contrast, cell density was normal in the mutant CA3. Furthermore, although the DG and CA3 layers were both thinner in P14 mutants compared to WT, the CA1 cell layer was of normal size. Putting these data together, the mutant DG had normal numbers of abnormally packed GCs, while mutant CA1 had significantly increased numbers of abnormally packed cells, and mutant CA3 contained significantly decreased numbers of normally spaced PyCs than WT mice.

This hippocampal pathology shifted as the animals matured. At P35, the 16GsoT/T DG and CA3 regions were not distinguishable from WT controls. However, while cell density had normalized in the mutant CA1, the PyC layer was significantly thinner than that of WT adult animals. Therefore, although other HC regions had normalized over time, in 16GsoT/T mutants there was a clear shift from increased numbers of CA1 PyC neurons at juvenile stages, to significantly decreased numbers of CA1 PyC in adults (Figure 4C, D).

These features are reminiscent of human neuropathology documented in association with ID, ASD, epilepsy, and other *AUTS2*-linked disorders (Varghese *et al.* 2017; Goodman *et al.* 2014; Otten and Meeter 2015; McKinnon *et al.* 2009). In the case of ASD, dense packing of hippocampal neurons has been linked to aberrant timing of neuronal maturation (Varghese *et al.* 2017; Amaral *et al.* 2008). Like the cerebellar abnormalities, these pathologies may be unique to 16Gso mutants since *Sox1* KO homozygotes display normal hippocampal structure (Malas *et al.* 2003), and hippocampal defects have not been reported for either *Auts2* KO mice or human patients to date.

### Global gene expression reveals region- and time-selective down-regulation of the Auts2 and Galnt17 genes

With the goal of gaining a clearer picture of genetic factors involved in these neuropathologies, we carried out global analysis of gene expression (RNA-seq) in CB and HC of 16GsoT/T mice and age-matched WT controls. For both regions, we examined gene expression at P14, when pathology is first observed in both brain regions, and at P35 to examine longer-term effects. Because male and female phenotypes were documented to be very similar (see above; Table S1) we focused all expression analyses on males **(**Table S2). In both brain regions, we saw differential expression of *Galnt17* and *Auts2* at one or both time points. In contrast, mis-expression of *Sox1* was not detected in either experiment. Results from each brain region are detailed below.

#### Cerebellum - dysregulation of extracellular matrix and signals of cellular stress:

In the P14 CB of 16GsoT/T animals, we saw significant down-regulation of *Galnt17* (-2.07 fold change) with no detectable effects on *Auts2* or *Sox1* (Table S2). Focusing more globally on the most highly differentially expressed genes (DEGs) in P14 CB (fdr < 0.05, ≥ 1.5 absolute value of FC), up-regulated DEGs were enriched in gene ontology (GO) functional categories related to corticotropin response and transcriptional activation, mostly related to immediate early genes (IEGs), such as *Fos*, that mark activated neurons (Table 2; Table S3**)**. P14 down-regulated DEGs were not enriched in any functional categories, but included genes associated with *AUTS2*-linked neurological disorders such as *Hif3a* (Zhang *et al.* 2014b), *Prodh*, *Hbegf* and *Fkbp5* (Yang *et al.* 2015; de Koning *et al.* 2015; Sasaki *et al.* 2015; Valdés-Moreno *et al.* 2017; Shao and Vawter 2008; Fani *et al.* 2013; Menke *et al.* 2013).

**Table 2:** DAVID Gene Ontology and Pathway analysis of DEGs (fdr0.05, ≥ 1.5FC) in HC and CB at P14 and P35

Brain region	Age	Functional Category
*Upregulated*		
cerebellum	P14	cellular response to corticotropin-releasing hormone stimulus
		positive regulation of transcription from RNA polymerase II promoter
cerebellum	P35	myelin sheath
		cellular response to interleukin-4
		growth cone
		neuron development neuron projection
		synaptic vesicle cycle
		unfolded protein binding
		calcium ion-regulated exocytosis of neurotransmitter
		axon guidance
		nervous system development
		inclusion body
		glucocorticoid receptor binding regulation of synaptic plasticity response to heat
hippocampus	P14	structural constituent of myelin sheath
		glycosaminoglycan biosynthesis - heparan sulfate / heparin
		cilium movement
		cilium organization
hippocampus	P35	response to cAMP
		cellular response to calcium ion
		amphetamine addiction Downregulated
cerebellum	P14	*no enriched categories*
cerebellum	P35	cell adhesion
		proteinaceous extracellular matrix
		ECM-receptor interaction
		growth factor binding
		cilium morphogenesis
		negative regulation of cell migration
hippocampus	P14	response to hypoxia
		response to amphetamine
		synaptic transmission, dopaminergic
hippocampus	P35	postsynaptic density
		glutamatergic synapse
		long-term potentiation response to cAMP
		cellular response to calcium ion

Representative categories are listed in order of their enrichment false discovery rate; for a full list of all enriched categories and statistics, see Table S4.

By P35, CB gene expression was much more dramatically dysregulated. *Galnt17* down-regulation was still observed but at a much lower level (-1.24 fold change); a slight up-regulation of *Auts2* (1.38 fold change) was also detected in CB at this later time point. More globally, and consistent with P14, P35 up-regulated DEGs were enriched in the GO category “cellular response to corticotropin-releasing hormone stimulus” as well as other signals of cellular stress, including “cellular response to interleukin-4”, “unfolded protein binding”, “response to heat” and “inclusion body”. Up-regulated P35 genes were also enriched in functions related to myelination, neurotransmitter exocytosis, axon guidance, synaptic plasticity and, neuron development (Table 2; Table S3).

P35 down-regulated CB DEGs were very strongly enriched in functions related to extracellular matrix but also categories such as cilium morphogenesis and motility, suggesting involvement of the ciliated ependymal cells (Table 2). Notably given that *Galnt17* functions as a GalNAcT, both CB DEGs included genes encoding enzymes central to protein glycosylation of various types (*Gcnt1*, *Extl1*, *Fut10*, *Ogt*, *Gcnt2*, *Man2c1*, *B3galnt2*, *B3galt2*, *B3gnt2*, *B4galtn4*, *B4galt1*, *Chpf2*, *Eogt*) and genes involved in downstream sulfation or sialylation of glycosylated sidechains (*Ndst3*, *St3gal6*, *St6galnac2*, *St8sia4*). The data suggested a broad dysregulation of glycosylation-related functions and a persistent signal of cellular stress. The significant up-regulation of genes related to neurodevelopment, growth cone, and synapses at P35 further suggested that in the mutant CB, neuron maturation occurs, but is significantly delayed.

#### Hippocampus - dysregulated neurotransmitter signaling and further signs of neuronal stress:

Looking next at HC datasets, we also observed significant *Galnt17* down-regulation at both time points in this region (-1.38 and -1.54 fold change at P14 and P35, respectively). In addition, we observed the down-regulation of *Auts2* transcripts, exclusively at P14 (measured collectively at -1.38 fold change). As in CB, *Sox1* was not among the DEGs at either time point. Globally, we identified certain DEGs and functions consistent with those identified in CB, but also clear evidence of unique HC effects. For example, genes including disease-related *Hif3a*, *Prodh*, *Fkbp5*, and *Hbegf*, were commonly down-regulated in P14 CB and HC, while others - including *Fos* - were consistently up-regulated (Table S2). Although at different ages (P14 in HC, P35 in CB), genes involved in oligodendrocyte development and myelination were also up-regulated in both brain regions. Cilia-related functions were also enriched in both brain regions although in opposite directions, being down-regulated in P35 CB but up-regulated in P14 HC. However, most enriched gene functions were brain region-specific. Most notably, genes involved in dopamine signaling - including the gene encoding dopamine receptor DRD2 - were significantly down-regulated in the HC of 16GsoT/T mice at the P14 time point (Table 2).

By P35, genes involved in neuron functions - including post-synaptic densities and long-term potentiation - were down-regulated in the 16GsoT/T HC. Genes related to glutamatergic signaling were also robustly and significantly down-regulated. Together with this signal of neurotransmitter imbalance, *Fos* and other IEGs were very highly up-regulated (Table S2). This expression pattern - echoing the upregulation of IEGs in mutant CB - strongly suggests a response to chronic neurological stress (Schreiber *et al.* 1991; Jett *et al.* 2017).

#### Brain region- and time-specific dysregulation of specific Auts2 isoforms:

Considering that the translocation could possibly dysregulate expression of *Auts2* isoforms differentially, we designed and calibrated primers to measure expression of the transcripts individually. In the present data set and in a previous project (Saul *et al.* 2017), we had noticed evidence for a novel *Auts2* promoter also included in a transcript model (ENSMUSG00000029673) and confirmed expression from this novel promoter by sequencing an qRT-PCR product from both CB and HC (Supplemental Text). We refer to this novel transcript as T6 (Figure 1B), and we also designed primers to detect its expression (Table S4).

**Figure 5:**
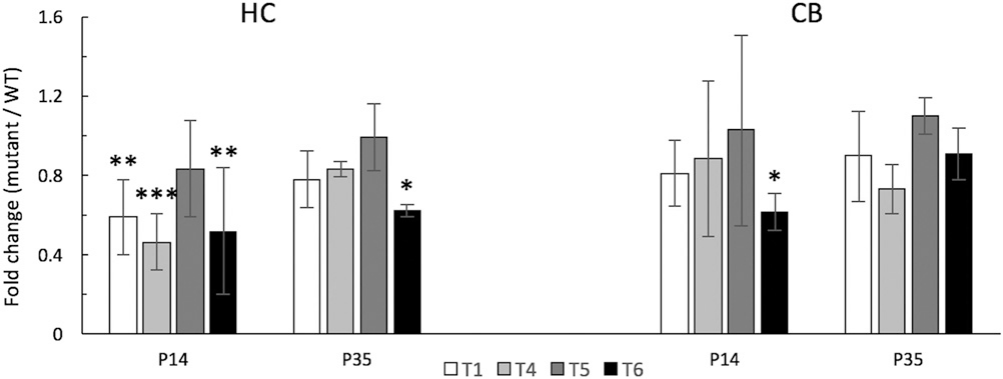
Cerebellar and hippocampal expression of *Auts2* transcripts in 16GsoT/T compared to WT controls at P14 and P35. *Auts2* isoforms (T1, T4, T5 and T6) expression in 16GsoT/T HC and CB at P14 and P35 was normalized to WT levels, which were set at 1. The expression of *Auts2* isoforms T1, T4 and T6 was decreased in P14 16GsoT/T HC, while in CB, only *Auts2* isoform T6 was significantly reduced. At P35, *Auts2* isoform T6 was significantly decreased in HC, and no *Auts2* isoform is dysregulated in CB. n = 3 for each genotype and sample. ANOVA, *P < 0.05; **P < 0.01; ***P < 0.001. CB = cerebellum; HC = hippocampus.

Differential expression of *Auts2* isoforms was detected by qRT-PCR in both brain regions, although in transcript-, region- and time-specific patterns. Transcripts T1 and T4 were both significantly down-regulated in P14 HC, whereas novel isoform T6 was down-regulated in both brain regions, with significant differences in HC at both P14 and P35 (Figure 5). In contrast, isoform T5 was not differentially expressed at either time point or brain region. We did not detect evidence of *Auts2* isoform up-regulation at P35 as suggested by RNA-seq data, possibly related to the relatively low levels of *Auts2* expression at the later time point and the small amounts of expression-level change. Nevertheless, the data confirmed that several *Auts2* transcripts are down-regulated by the 16Gso mutation during early postnatal development, with peak effects around P14.

### Auts2 and Galnt17 are co-expressed in a broad array of brain cell types

These data suggested primary roles for *Auts2* and *Galnt17* in the postnatal development of 16GsoT/T brain pathologies. To further assess these potential roles, it was important to document the cell types in which AUTS2 and GALNT17 proteins are expressed. *Auts2* expression has been documented in detail: the gene is highly expressed in both cerebellar PCs and hippocampal granule and pyramidal cells in adults, with a highly dynamic pattern across the brain during development (Bedogni *et al.* 2010). However, brain expression or function has never been investigated for *Galnt17*.

**Figure 6:**
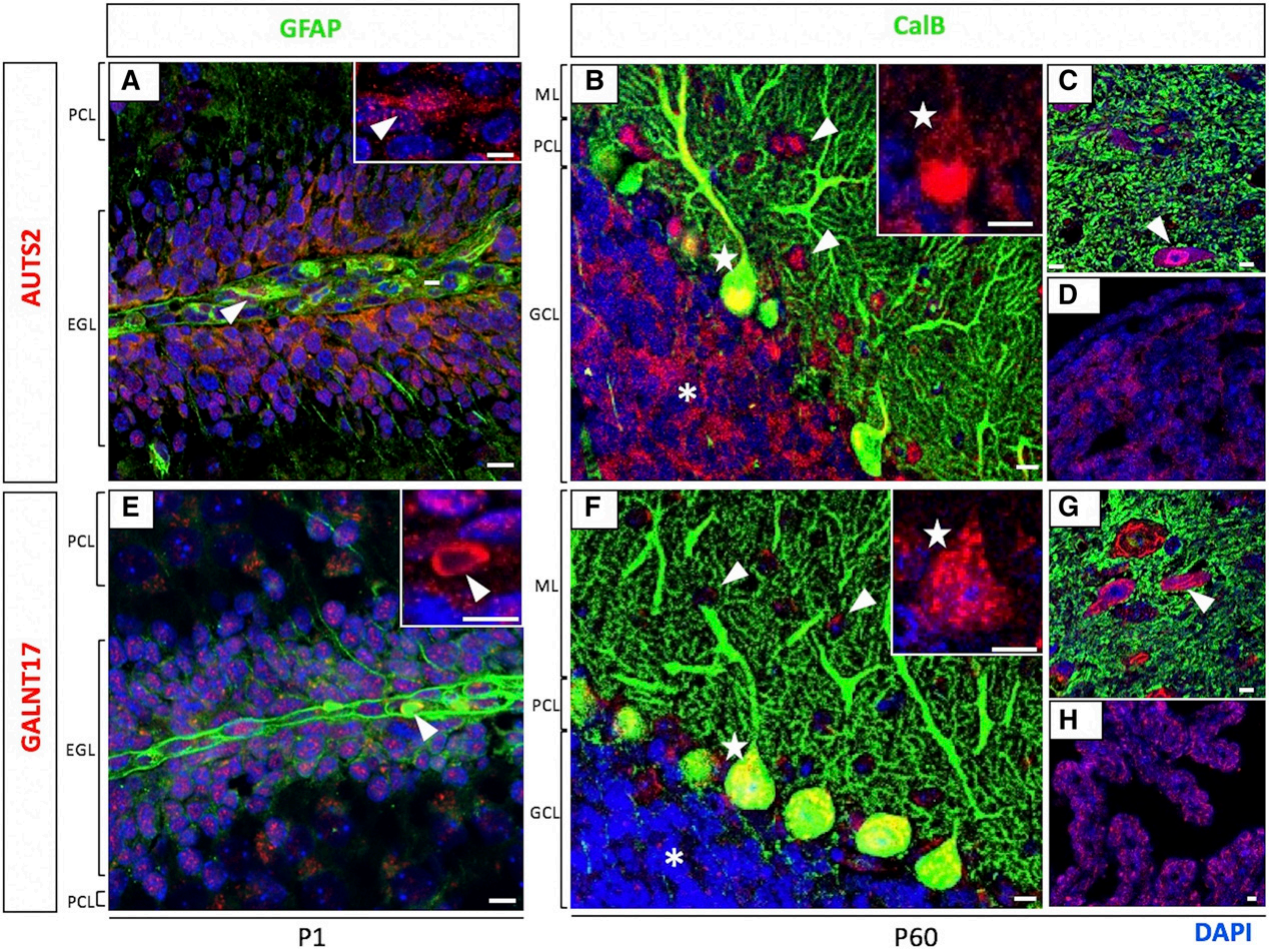
Co-expression of AUTS2 and GALNT17 in different cell types in CB. Expression of AUTS2 (red; top) and GALNT17 (red; bottom) in various cell types was determined by co-staining with antibodies to different markers, and by cell shape and location in CB. Both proteins are expressed in Bergmann glia (A and E; arrowheads) as marked by GFAP in green at P1, and in PCs (B and F; stars) as marked by Calbindin (CalB) in green at P60. Both proteins were also co-expressed in cerebellar GCs (B and F; asterisks), stellate cells (B and F; arrowheads), large neurons in the deep cerebellar nuclei (C and G; arrowheads), and choroid plexus (D and H). scale bar = 10 μm. PCL = Purkinje cell layer; EGL = external granule layer; ML = molecular layer; GCL = granule cell layer.

To examine protein expression, we used IHC with antibodies to AUTS2 and GALNT17 in CB and HC sections from WT animals. As revealed by these experiments, AUTS2 and GALNT17 proteins are both highly expressed in PCs, co-localizing with Calbindin (CalB) in juvenile (not shown) and adult brain sections (stars in Figure 6B, F**)**. We also saw co-expression in the smaller CalB-negative basket and stellate cells nestled among the PC dendrites, with AUTS2 expressed in the nucleus and GALNT17 expressed in the cytoplasm, respectively (arrowheads in Figure 6B, F). AUTS2 and GALNT17 were also detected in the cytoplasm of the cerebellar granule cells (asterisks in Figure 6B, F). We noted three other co-expression sites in the cerebellar region. First, as reported previously (Bedogni *et al.* 2010), AUTS2 was detected in selected neurons within the deep cerebellar nuclei; GALNT17 was also detected at high levels in these cells (arrowheads in Figure 6C, G). Also, consistent with previous data on AUTS2, both proteins were detected in the choroid plexus and ependymal cells (Figure 6D, H). Particularly relevant to the 16Gso cerebellar pathology but not previously reported, both AUTS2 and GALNT17 were also detected in Bergmann glia, as confirmed by co-localization with marker GFAP in Bergmann glia cell bodies at P1 (arrowheads in Figure 6A, E). Notably, every cell type that we found to express AUTS2 also expressed GALNT17.

**Figure 7:**
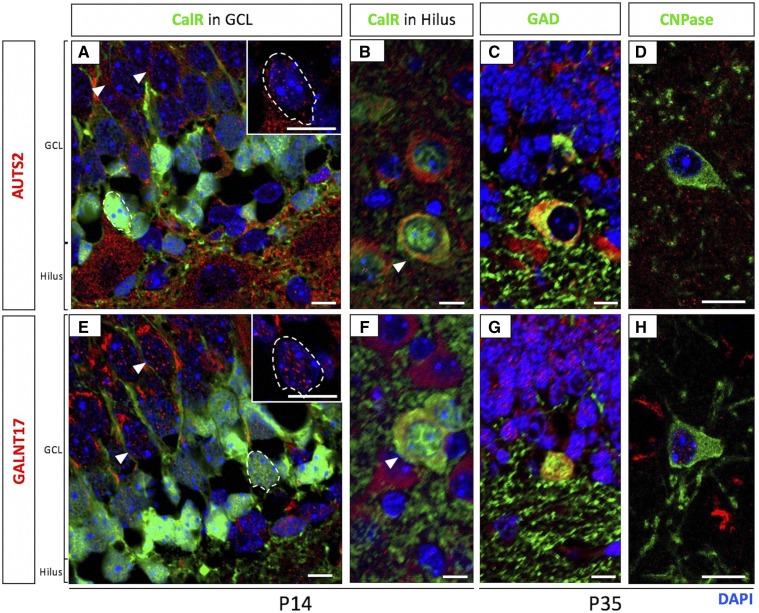
Co-expression of AUTS2 and GALNT17 in different cell types in HC. Expression of AUTS2 (red; top) and GALNT17 (red; bottom) in various cell types was determined by co-staining with antibodies to different markers, by cell shape, and by location in HC. Both proteins are expressed in immature GCs (A and E; dashed circles) marked by Calretinin (CalR) and in mature GCs (A and E; arrowheads), as well as mossy cells (B and F; arrowheads) marked by CalR, GABAergic pyramidal basket cells (C and G) marked by GAD 65/67, and oligodendrocytes (D and H) marked by CNPase. Scale bar = 10 μm. GCL = granule cell layer.

In postnatal HC, AUTS2 protein has been reported to co-localize with markers of Calretinin (CalR)-expressing immature granule neurons in the subgranular zone (SGZ) and granule cell layer (GCL) of the dentate gyrus (DG) as well as mature GCs in the GCL (Bedogni *et al.* 2010). We confirmed AUTS2 and demonstrated GALNT17 protein expression in both cell populations at P14 as well (Figure 7A, E). In mature GC, the GALNT17 protein was concentrated into bright punctate foci (Figure 7E, arrows**)**, consistent with previous reports of the protein’s concentration within the Golgi apparatus (Nakayama *et al.* 2012). However, in immature cells in the SGZ, the GALNT17 signal was more evenly distributed including staining in the nucleus (Figure 7E, dashed circles). These cells were also CalR+, confirming that GALNT17 and AUTS2 are co-expressed GC from a very early stage in their differentiation (dashed circles in Figure 7A, E). AUTS2 and GALNT17 proteins were also found together in PyC of the CA1 and CA3 regions of the HC (not shown). Additionally, we saw expression of both proteins in GABA-ergic (GAD 65/67 positive, or GAD+) cells, including the pyramidal basket cells located at the edge of the DG (Figure 7C, G) and scattered cells inside the DG hilus. Inside the ventral HC hilus, we also detected expression of both proteins in the large, scattered CalR+ mossy cells (Figure 7B, F, arrowheads). We further detected expression of both AUTS2 and GALNT17 proteins colocalizing with the marker CNPase (Figure 7D, H), indicating expression in oligodendrocytes, which has not been reported for AUTS2 previously. Therefore, many of the cell types thought to be abnormal based on RNA-seq experiments – including neurons as well as oligodendrocytes and ependymal cells – co-express AUTS2 and GALNT17.

### Leveraging clues from transcriptomic data for new mechanistic insights

Based on RNA-seq data, we hypothesized that *Galnt17* LOF and global dysregulation of glycosylation is a major contributor to the unfolded protein response and cellular stress, the delayed differentiation of PCs, and failed development of Bergmann glia in mutant mice. We conjectured that the 16GsoT/T hippocampal pathology could also reflect abnormal developmental timing based on several bits of evidence. For example, RNA-seq data suggested delays in differentiation of neuronal intermediate progenitor cells (IPCs), based on down-regulation of markers of later differentiation stages - including *Neurod1* and *Prox1* (later intermediate progenitors), a post-mitotic marker for immature GC, *Calb2* (encoding CalR), and mature GC marker *Calb1* (CalB) (Table S3). Furthermore, as mentioned above, the abnormally dense packing of GCs and PyCs we observed is reminiscent of pathologies observed in the HC of ASD patients where it is thought to reflect abnormal timing of neuron maturation, dendrite formation, and synaptic connectivity (Amaral *et al.* 2008; Varghese *et al.* 2017). Importantly, mis-timed maturation of hippocampal neurons could potentially explain key 16GsoT/T behavioral phenotypes -including impaired learning and memory as well as seizures – as well as related human AUTS2 syndrome phenotypes (Beunders *et al.* 2013).

**Figure 8:**
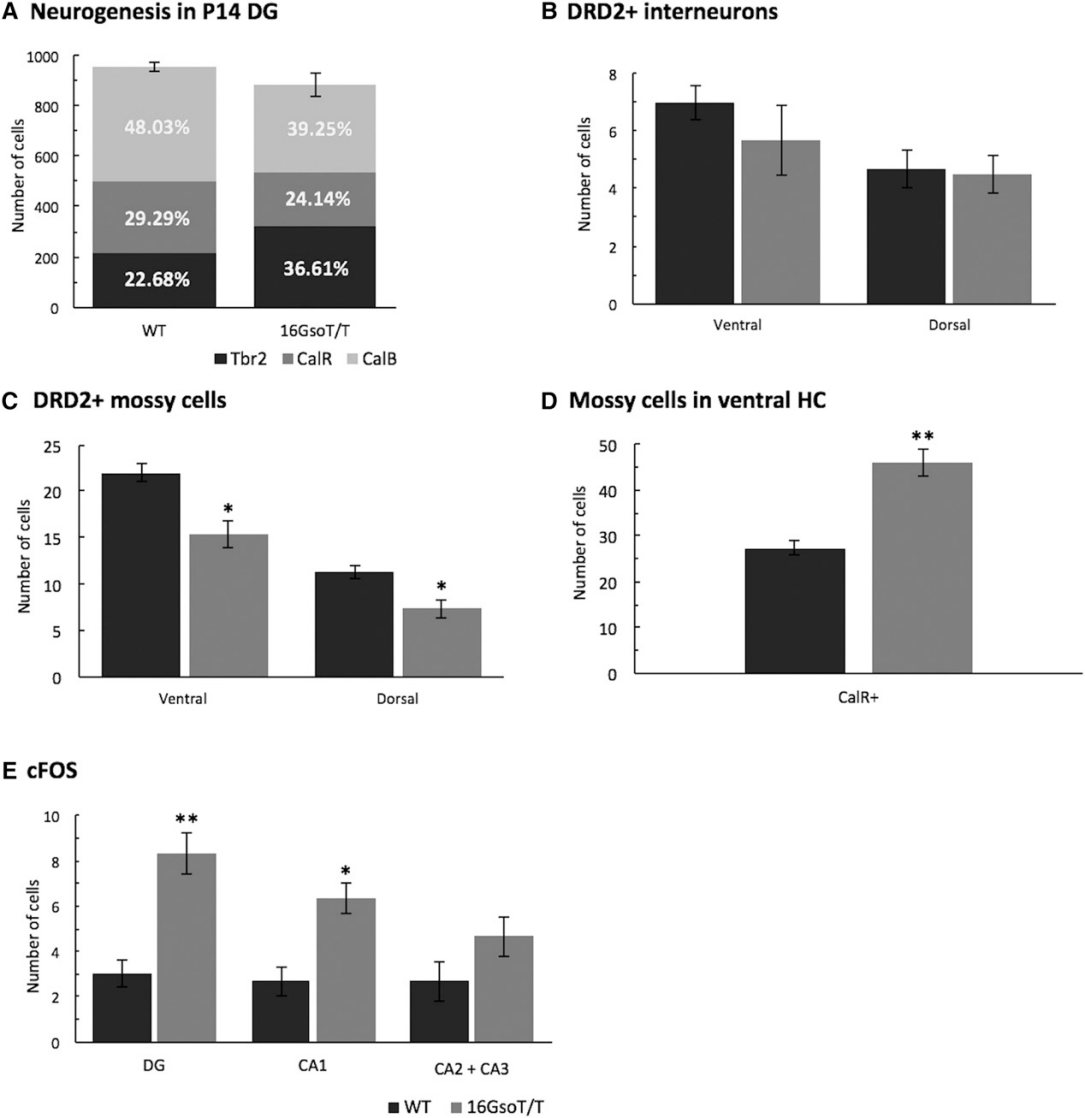
Molecular confirmation of 16GsoT/T hippocampal pathologies. (A) Percentage of IPCs (Tbr2+), immature GC (CalR+), and mature GC (CalB+) were measured in the DG at P14, with 16GsoT/T (gray bars) compared to age-matched WT controls (black bars). 16GsoT/T animals showed increased numbers of Tbr2+ IPCs (36.61% of the total numbers of neurons), but decreased numbers of immature (24.14%) and mature (39.25%) GC compared with WT (black). However, there was no difference in total number of cells in WT and 16GsoT/T hippocampal regions examined. (B) Number of DRD2+, GAD+ interneurons in ventral and dorsal HC. We detected no significant difference in numbers of interneurons between WT (black bars) and 16GsoT/T (gray bars) P14 animals. (C) Number of DRD2+ mossy cells in ventral and dorsal HC. Our counts revealed that 16GsoT/T animals have significantly fewer DRD2+ mossy cells in both dorsal and ventral HC than WT controls. (D) Number of overall mossy cells are significantly more in 16GsoT/T than in WT in ventral HC. (E) Number of cFOS+ cells in DG, CA1, and CA2 and CA3 hippocampal regions at P35. 16GsoT/T P35 HC contained more cFOS+ cells in DG and CA1 regions than in WT controls, but similar numbers in CA2/3. n = 3 for both genotypes. DG = dentate gyrus; HC = hippocampus. ANOVA, *P < 0.05; **P < 0.01.

To address this possibility, we examined the developmental trajectory of DG GCs, which are marked by location and well-established markers (reviewed by (Khalaf-Nazzal and Francis 2013; Nicola *et al.* 2015)). We used antibodies to TBR2, CalR, and CalB to identify and count the numbers of cells of each type in the DG of P14 mutants and controls. Whereas the DG of P14 16GsoT/T and WT mice contain similar numbers of neurons overall (Figure 8A), P14 mutants contain higher relative numbers of TBR2+ IPCs and fewer CalR+ and CalB+ cells than controls (Figure 8A). By P35, the numbers of TBR2+, CalR+ and CalB+ neurons in mutant DG had normalized (not shown) along with cell density (Figure 4C, D). This finding is consistent with the idea that in 16GsoT/T mutants, the dense packing of GC at P14 is a signature of the cells’ immaturity, and that hippocampal GC differentiation is significantly delayed.

A second transcriptomic signal we sought to investigate was the reduced dopaminergic signaling detected in the P14 16GsoT/T animals – centered around the dopamine receptor type 2 gene, *Drd2*. In the HC, *Drd2* is expressed primarily in DG hilar mossy cells and interneurons, both of which interact intimately with and regulate hippocampal GC and PyC. To identify the cell types responsible for reduced DRD2 expression, we stained P14 16GsoT/T and WT hippocampal sections with a DRD2 antibody together with markers for interneurons (GAD+) to clearly distinguish them from mossy cells. The results revealed that DRD2+, GAD+ interneurons were present in normal numbers in 16GsoT/T mutant mice (Figure 8B**)**. In contrast, DRD2+, GAD- mossy cells were present in significantly lower numbers in 16GsoT/T mutant dorsal and ventral HC compared to WT controls (Figure 8C). However, when we used CalR to identify hilar mossy cells independently of the DRD2 signal, we saw that the mossy cells were actually present in higher numbers in 16GsoT/T than in WT animals in ventral HC (Figure 8D). Therefore, mossy cells are generated in higher numbers, and migrate properly to the hilus in 16GsoT/T HC, DRD2 protein is expressed at significantly reduced levels in that population of cells.

Finally, we used an antibody to cFOS to identify the sources of the increased IEG expression in 16GsoT/T HC and CB. In P35 HC, increased cFOS signal was detected clearly in the DG GCL (Figure 8E) and in CA1 PyCs. However, we found no significant difference in cFOS signals between 16GsoT/T mutants and WT in CA2 or CA3 regions. Together with the histopathological findings, these data suggest that 16GsoT/T hippocampal pathology is primarily focused in the DG and CA1 regions, and that despite the normalized cell numbers in adult DG, cellular pathology persists in both regions into adulthood.

## Discussion

Here we provide a detailed molecular, behavioral, cellular, and neuropathological analysis of mice inheriting T(5G2;8A1)GSO (16Gso), a novel reciprocal translocation involving chr5 and chr8. On chr5, the breakpoint falls in the intergenic region between *Galnt17* and *Auts2*; the translocation affects expression of *Galnt17* as well as specific *Auts2* alternative promoters in a conditional manner, with distinct spatial and temporal effects on each. Importantly, this dysregulation was detected in brain regions that show distinct neuropathologies in 16GsoT/T mice and at the developmental time points during which these pathologies first appear. Although *Galnt17* mutations have not been investigated, several phenotypes detected in 16GsoT/T mice overlap obviously with those of *Auts2* KO mutants, and share features with human AUTS2 syndrome patients as well (Table 1).

On chr8, the 16Gso translocation breakpoint falls upstream of the *Sox1* gene; mis-expression of *Sox1* was not detected in the brain regions or postnatal time points we examined, but *Sox1* KO homozygotes display seizures, freezing, and anxiety-related behaviors also expressed by 16GsoT/T mice (Table 1). Although we cannot rule out a subtle role for this gene in the 16Gso behavioral phenotypes, several lines of evidence argue against *Sox1* as a central driver. In particular, neither *Sox1* KO homozygotes (Malas *et al.* 2003; Sottile *et al.* 2006) nor 16Gso/+, *Sox1/+* double heterozygotes (Weisner 2015) display the distinctive brain pathologies that we detected in 16GsoT/T mice. Moreover, genetic complementation tests between the recessive 16Gso and *Sox1* KO alleles have been completed, and neither brain pathologies, seizures nor exaggerated freezing behaviors were observed in 16Gso/+, *Sox1*^-/+^ compound heterozygotes (Weisner 2015).

Here we should note that epilepsy is one of the phenotypes shared by 16Gso mutants and some AUTS2 syndrome patients (Table 1), suggesting that even this phenotype could be related to chr5 breakpoint-spanning genes. However, the specific hippocampal and cerebellar pathologies we identified in 16GsoT/T mice have not been previously associated with any *Auts2* mouse mutations and other than generalized microcephaly, specific brain pathologies have not been described for human AUTS2 patients to date. On the other hand, severe cortical abnormalities that have been documented for *Auts2* KO homozygotes (Hori *et al.* 2014) are not seen in 16GsoT/T mice. We hypothesize that these differences in behavior and pathology are likely due to the translocation’s partial, isoform-specific, and spatially- and temporally-limited effects on *Auts2* expression, with a possible contribution from the coordinated dysregulation of *Galnt17*.

Testing these conjectures will require further investigation with conditional mutants, including a first look at the brains and behaviors of *Galnt17* mice. Nevertheless, the unique 16GsoT/T cerebellar and hippocampal pathologies - including the mistiming of both PC and hippocampal neuron differentiation and PC loss – hold significant potential for human clinical relevance. These 16GsoT/T phenotypes bear some very striking similarities to those observed in ASD patients (Amaral *et al.* 2008; Varghese *et al.* 2017; Fatemi *et al.* 2002; Courchesne and Pierce 2005; Courchesne *et al.* 2005), and related cellular phenotypes have been tied to temporal lobe epilepsy, bipolar disorder, depression, schizophrenia, and ADHD as well (Chambers 2013; Goodman *et al.* 2014; McKinnon *et al.* 2009; Otten and Meeter 2015; Hester and Danzer 2014; Finch *et al.* 2002; Maloku *et al.* 2010; Strata 2015). The similarities suggest a broad relevance to *AUTS2*-linked disease.

Importantly, 16Gso neuropathologies involve cell populations that express AUTS2 and GALNT17 during the critical postnatal period when the pathologies first appear. Indeed, we show that the two proteins are highly co-expressed in brain; in fact, we could not identify a single cell type in which one was expressed and the other was not. One possible explanation for this tight co-expression could be that *Galnt17* is a target of AUTS2 nuclear isoforms; however, arguing against this possibility, *Galnt17* binding has not been indicated by AUTS2 chromatin precipitation to date (Oksenberg *et al.* 2014), and we found *Galnt17* to be highly down-regulated in P14 CB in the absence of effects on *Auts2*. Rather, we posit, the high level of co-expression suggests that the two genes are controlled within a common three-dimensional regulatory structure, perhaps involving interactions with shared local enhancers. This possibility is supported by the fact that three-dimensional interactions between *Auts2* and *Caln1* (located directly downstream of *Galnt17*) are regulated by cocaine in striatal spiny neurons (Engmann *et al.* 2017). Furthermore, the cellular functions attributed to GALNT17 – ECM interactions, cell adhesion, cell motility, formation of lamellipodia and filopodia, and endocytosis (Nakayama *et al.* 2012) - dovetail well with those identified for AUTS2 (Hori *et al.* 2014); these data suggest that the proteins could be coordinated to act in the same pathways in developing neuronal cells.

The current study provides novel mechanistic clues to these pathways and possible roles of each gene. For example, RNA-seq data implicate *Galnt17* LOF as the major contributor to the delayed maturation and age-related loss of PCs, whereas reduced expression of *Auts2*, *Galnt17*, or both genes in concert could contribute to the 16GsoT/T hippocampal pathology. Interestingly, common themes of delayed neuronal maturation and cellular stress were detected in both brain regions. Connecting brain pathology to behaviors, we surmise that the abnormal development of hippocampal neurons is linked to 16GsoT/T learning deficits, and suggest that similar pathologies may be relevant to ID in AUTS2 syndrome patients as well. Furthermore, the failure of hippocampal connectivity, PC loss, or a combination of the two could contribute to developmental delay, anxious and repetitive behaviors, and seizures. The present data can be used to design experiments involving strategic combinations of conditional mutants together with new tools, such as single-cell sequencing, to address these hypotheses.

As mentioned, genotype-phenotype relationships in the *AUTS2* region are complicated, as has been studied and summarized in great depth by expert groups (Beunders *et al.* 2016; Beunders *et al.* 2013; Oksenberg and Ahituv 2013). Our data suggest that different combinations of *AUTS2* isoform mis-expression, perhaps combined with effects on co-regulated *GALNT17*, could explain some of the phenotypic heterogeneity. Interestingly, in addition to a multigenic and *Auts2* intragenic deletions and duplications, human duplications contained completely within the intergenic region between *AUTS2* and *GALNT17* have been reported in public databases, and are associated with generalized developmental delay (for example, in the ClinVar database (Landrum *et al.* 2016)). Data from the 16Gso translocation would predict that these and perhaps other noncoding variants in the region could impact expression of both genes. However, gene expression has not been investigated in conjunction with any AUTS2 syndrome mutations; fortunately, questions raised by this study can be addressed by further studies in mouse and other model organisms.

It is now well-established that even small, intragenic mutations can disrupt enhancers and impact expression of neighboring genes (Dixon *et al.* 2015; Dixon *et al.* 2012; Griffin *et al.* 2002; Kleinjan *et al.* 2006; Kleinjan and Coutinho 2009), and we propose that a clear picture of genotype-phenotype associations in this region will emerge with incorporation of a detailed regulatory map. An elegant study in zebrafish has identified several conserved *Auts2*-region enhancers (Oksenberg and Ahituv 2013), but additional regulatory elements active in specific brain cell types and at additional developmental time points are certain to emerge. Fortunately, tools for investigating genome-wide regulatory relationships are becoming increasingly accessible, and can possibly provide key missing pieces to this genetic puzzle.

Since *Galnt17* expression and *in vivo* functions have not previously been characterized, our data, revealing the protein’s broad co-expression with AUTS2 and its potential role in regionally-associated neuropsychiatric disorders, are both novel and significant. This study builds groundwork for understanding the neurodevelopmental underpinnings of AUTS2 syndrome behavioral phenotypes and of other types of regionally-associated neurological disease, and suggests roles for each gene in these phenotypes. More generally, this study raises the possibility that other clusters of topologically-linked, coregulated genes could explain natural variance in expression many types of neurodevelopmental disorders, including those associated with mutations within the boundaries of well-studied genes.
